# Antitumoral Properties of Natural Products

**DOI:** 10.3390/molecules25030650

**Published:** 2020-02-03

**Authors:** Roberto Fabiani

**Affiliations:** Department of Chemistry, Biology and Biotechnology, University of Perugia, 06126 Perugia, Italy; roberto.fabiani@unipg.it

Cancer is one of the major causes of death worldwide. It is a multifactorial heterogeneous disease characterized by a multistep process initiated by genetic alterations of normal cells which become malignant cells. These cells are characterized by uncontrolled cell growth, immortality, invasiveness, and ability to form distant metastasis. Natural bioactive molecules may interfere with the carcinogenesis process by altering the tumor cell behavior and targeting several molecular abnormally activated signaling pathways.

Among different cancer types, glioblastoma is the most frequent and aggressive of all malignant brain tumors. Glioblastoma is highly invasive, and its treatment include surgery, radiation, and chemotherapy with temozolomide (TMZ). Nevertheless, patient prognosis remains poor and associated with a low survival rate. In this Special Issue, Franco et al. have investigated the anticancer properties of coronarin D, a diterpene isolated from a dichloromethane extract of *Hedychium coronarium* in a glioblastoma cell line (U-251) [1]. They found that this compound was able to inhibit proliferation and induce G1 cell cycle arrest and apoptosis in U-251 cells. The authors proposed that coronarin D-induced effects were mediated by an overproduction of reactive oxygen species, which promoted phosphorylation of H2AX and ERK, increased the expression of p21, and activated caspases. Noteworthy is the observation that coronarin D was in some cases even more effective than TMZ. Similarly, Silva et al. have demonstrated that ingenol-3-dodecanoate (IngC), a semi-synthetic ingenol derivative from *Euphorbia tirucalli*, was more effective at inhibiting the growth of different glioma cell lines than TMZ [2]. Overexpression of p21 was also observed in both GAMG and U373 glioblastoma cell lines. On the other hand, IngC promoted S-phase arrest but was not able to induce apoptosis. Worth noting is the observation that IngC promoted glioma cell autophagy as evidenced by both the accumulation of LC3B-II and the development of acidic vesicular organelles. In addition, IngC treatment resulted in potent inhibition of protein kinase C activity, showing differential actions against various PKC isotypes [2]. Hong at al. demonstrated a cytotoxic effect of SB365, a saponin D extracted from the roots of *Pulsatilla koreana,* on U87-MG and T98G glioblastoma multiforme (GBM) cells [3]. This compound induced caspase-independent cell death, inhibited autophagic flux, and deteriorated lysosomal stability and mitochondrial membrane potential (MMP) in U87-MG cells. Very importantly, this paper showed also an additive effect between SB365 and TMZ on glioblastoma cell proliferation both in vitro and in vivo using a mouse U87-MG xenograft model [3]. Furthermore, Bonturi et al. have studied the effect of a plant-derived protein obtained from *Crataeva tapia* tree bark lectin (CrataBL) on U87 glioblastoma cells in co-culture with mesenchymal stem cells [4]. They showed that the mixed cells grown in 1:1 co-culture were more sensitive to the CrataBL than each of the individual cell types with regards to both inhibition of proliferation and induction of death. Corrêa et al. have developed and characterized liposomal nanocapsules loaded with purified tarin, a lectin naturally found in taro corms (*Colocasia esculenta*), which were able to dose-dependently inhibit glioblastoma (U-87 MG) and breast (MDA-MB-231) tumor cells, while free tarin did not affect tumoral cell growth [5]. Finally, a study by Pham et al. has investigated the effects of neferine on another brain tumor, neuroblastoma [6]. They showed that neferine suppressed proliferation and induced both apoptosis and autophagy in human neuroblastoma IMR32 cells [6].

Osteosarcoma (OS) is one of the most frequent bone tumors, with a high prevalence in teenagers and young adults. Due to its high metastatic potential, the prognosis of osteosarcoma patients is poor. Although the main treatment is surgery, anticancer strategies based on development of new agents that target particular intracellular signaling pathways in osteosarcoma cells are needed. The study of Yang et al. has shown that CLEFMA (4-[3,5-bis(2-chlorobenzylidene)-4-oxo-piperidine-1-yl]-4-oxo-2-butenoic acid), a synthetic analog of curcumin, decreased viability and induced apoptosis in human osteosarcoma U2OS and HOS cells [7]. This compound activated both extrinsic and intrinsic apoptotic pathways and, by using specific inhibitors, the authors demonstrated that these effects were mediated by the phosphorylation of c-Jun N-terminal kinases (JNK) and p38, while the ERK pathway was not involved [7]. Diallyl disulfide (DADs), a natural organic compound present in garlic and scallion, inhibited proliferation, induced apoptosis, and arrested the cell cycle at the G2/M phase of MG-63 osteosarcoma cells [8]. In addition, DADs induced the formation of autophagosome as revealed by the increased expression of LC3-II protein which was reduced by the autophagy inhibitor 3-methyladenine. These anticancer effects were mediated by the ability of DADs to inhibit the PI3K/Akt/mTOR signaling pathway [8]. Similar to DADs, Shen et al. showed that licochalcone A, a flavonoid extracted from licorice root, reduced proliferation, caused G2/M phase cell cycle arrest, and induced apoptosis and autophagy in HOS and MG-63 osteosarcoma cells [9]. Furthermore, it was found that licochalcone A induced rapid phosphorylation of Chk2 and ATM, suggesting that the ATM–Chk2 pathway may contribute to its effect on G2/M phase arrest. By using the autophagy inhibitor chloroquine, it was also demonstrated that the observed autophagy was associated with licochalcone A-induced apoptosis [9]. Hsu et al. have reported that coronarin D reduced the proliferation of HOS and MG-63 osteosarcoma cells while had a minor cytotoxic effect in human fibroblasts (MRC-5) [10]. This compound induced apoptosis and mitotic phase arrest in osteosarcoma cells and JNK was found to play a crucial role in coronarin D-induced effects [10].

Lung cancer is the leading cause of cancer incidence and mortality worldwide, with 2.1 million new cases and 1.8 million deaths predicted in 2018. The high mortality and low survival rate, that have remained unchanged in recent years, support the efforts to find new antineoplastic drugs to overcome this malignancy. In the present Special Issue, four papers have described the anticancer activity of different natural products on lung cancer cell in vitro [11,12,13,14]. Ooppachai et al. showed that dicentrine, an aporphine alkaloid found in the roots of *Lindera megaphylla* and several other plants, potentiated the TNF-induced apoptosis in A549 lung adenocarcinoma cells [11]. This compound was also able to inhibit the TNF-induced invasion, migration, and expression of metastasis-associated proteins. These effects were due to, at least in part, to the suppression of TAK-1, MAPK, Akt, AP-1, and NF-kB signaling pathways [11]. Similar effects were induced by treatment of A549 lung adenocarcinoma cells with a proanthocyanidin-rich fraction obtained from red rice [12]. Similarly, antrodin C (ADC), a maleimide derivative isolated from mycelium of *Taiwanofungus camphoratus*, exerted its anticancer activities (antiproliferative and pro-apoptotic) by suppressing both the Akt/mTOR and AMPK signaling pathways in human lung adenocarcinoma cell line SPCA-1 [13]. Importantly, this study showed that ADC was not toxic toward normal human lung cells BEAS-2B [13]. Cucurbitacin B (CuB) is a natural tetracyclic triterpenoid compound mainly found in Cucurbitaceae. The study of Liu et al. reported, for the first time, that CuB induced EGFR degradation and inhibited CIP2A/PP2A/Akt activities in different in gefitinib-resistant non-small cell lung cancer cell lines [14].

After lung cancer, breast cancer is the second main cause of death by cancer in women. Triple-negative breast cancer (TNBC), characterized by the absence of expression of three receptors (estrogen, progesterone, and human epidermal growth factor receptor 2), is an aggressive type of breast cancer that is difficult to treat. Jin et al. have reported the antitumoral properties of 13-ethylberberine (13EBR), a derivative of berberine (alkaloid isolated from *Cotridis rhizoma*), on MDA-MB-231 cells, which are a common model for TNBC [15]. 13-EBR reduced proliferation and induced apoptosis in both MDA-MB-231 and radiotherapy-resistant RT-R MDA-MB-231 cells. These effects were mediated by the promotion of reactive oxygen species production and regulation of the apoptosis-related proteins involved in the intrinsic pathway [15]. In the same cell model, Tian et al. showed antitumor activity of 4-epi-isoinuviscolide (CLE-10), a sesquiterpene lactone isolated from *Carpesium abrotanoides* L. [16]. This compound induced pro-death autophagy and apoptosis in MDA-MB-231 cells by upregulating the protein expressions of LC3-II, p-ULK1, Bax, and Bad, and downregulating p-PI3K, p-Akt, p-mTOR, p62, Bcl-2, and Bcl-xl [16]. Tan et al. have used another model of breast cancer (MCF-7 cells) to study the antiproliferative activity of the water soluble natural yellow *Monascus* pigments [17]. These compounds reduced the migration and invasion of MCF-7 cells, and these activities were associated with a downregulation of the expression of matrix metalloproteinases and vascular endothelial growth factor [17].

Gastric cancer is the fourth most common cancer and the second leading cause of cancer death worldwide. Several studies have been performed to find new therapeutic strategies based on bioactive phytochemicals with a lower toxicity. Zeylenone (Zey), a cyclohexene oxide isolated from the leaves of *Uvaria grandiflora*, has shown multiple anticancer activities on different cell lines. In their study, Yang et al. have demonstrated that this compound was able to induce mitochondrial apoptosis and to inhibit migration and invasion in SGC7901 and MGC803 gastric cancer cells in vitro [18]. In addition, Zey downregulated the expression of matrix metalloproteinase 2/9 and inhibited the phosphorylation of AKT and ERK. Of particular interest is the in vivo observation that Zey effectively inhibited tumor growth in nude BALB/c mice bearing BGC823 xenografts without any evident cytotoxic effects [18]. Bo et al. have investigated the anticancer activity of bovine lactoferrin hydrolysate (BLH) with added Cu^2+^ and Mn^2+^ on human gastric cancer BGC-823 cells [19]. They showed that the fortified BLH products had higher activities than BLH alone as evidenced by induction of apoptosis and activation of the classic caspase-3-dependent apoptotic pathway [19]. As opposed to the use of single purified molecules, several studies have highlighted the anticancer effects of plant-derived fractions and/or extracts on different cell models. Gomes at al. have demonstrated a cytotoxic effect of different *Annona coriacea* Mart. derived fractions on cisplatin-resistant cervical cancer cell lines (CaSki, HeLa, and SiHa) and on a normal keratinocyte cell line (HaCaT) [20]. Lin et al. have investigated the tumor-suppressive effects of an ethanol extract from *Paris polyphylla* in DLD-1 human colorectal carcinoma cells [21]. They found that cell death was induced by the upregulation of autophagy markers and treatment in combination with doxorubicin enhanced its cytotoxicity (12). Wei et al. have studied the anticancer activity of an ethanol extract from *Artemisia absinthium* and some subfractions on hepatocellular carcinoma cells [22]. The results showed the inhibition of cells growth and induction of apoptosis which might be mediated by the endoplasmic reticulum stress and mitochondrial-dependent pathway [22]. In addition, it was demonstrated an inhibition of tumor growth in vivo using the H22 tumor mouse model (H22 cells were subcutaneously injected in male Kunming mice and tumor sizes were monitored over time). Interestingly, the extract improved the survival of tumor mice without obvious toxicity and side effects [22]. Willer et al. have assayed extracts and fractions derived from damiana (*Turnera diffusa*) against different myeloma cell lines (NCI-H929, U266, and MM1S) [23]. They identified the flavanone naringenin as the most active compound able to decrease viability in particular in NCI-H929 cells. Furthermore, apigenin 7-O-(4”-O-p-E-coumaroyl)-glucoside was identified as being cytotoxic for the first time. This study also described the first validated UHPLC-DAD method for the quantification of phenolic constituents in *Turnera diffusa* [23]. Huang et al. have found that a *Ganoderma tsugae* ethanol extract, a Chinese natural and herbal product, significantly inhibited expression of SREBP-1 and its downstream genes associated with lipogenesis in prostate cancer cells (LNCaP and C4-2) [24]. These effects were associated to the inhibition of cell growth, migration, and invasion, and induction of apoptosis [24]. Ferhi et al. have shown the antiproliferative effects ethanol and water extracts from grape leaves on HepG2 hepatocarcinoma, MCF-7 human breast cancer cells, and vein human umbilical (HUVEC) cells [25]. In cancer cells, both extracts induced the expression of the pro-apoptotic gene Bax and reduced the expression of the anti-apoptotic gene Bcl-2. Interesting, the extracts did not show toxic effects on vein umbilical HUVEC cells [25]. Elansary et al. have characterized the phenolic profiles of *Catalpa speciosa*, *Taxus cuspidata*, and *Magnolia acuminata* bark extracts and studied their antiproliferative activity against different cancer cell lines (MCF-7, HeLa, Jurkat, T24, and HT-29) [26]. Yang et al. have screened 11 different lichen acetone extracts on the stemness potential of colorectal cancer cells and have isolated the most active compound tumidulin from *Niebla* sp. [27]. This compound reduced spheroid formation and the mRNA expression and protein levels of different cancer stem markers (ALDH1, CD133, CD44, Lgr5, and Musashi-1) in CSC221, DLD1, and HT29 cells [27]. Alvarado-Sansininea et al. have isolated quercetagetin and patuletin from *Tagetes erecta* and *Tagetes patula* flower ethanol extracts and tested for their antiproliferative, necrotic, and apoptotic activity on different cancer cell lines (CaSki: cervical, MDA-MB-231: breast, SK-LU-1: lung) [28]. The structure–activity relationship study, including also quercetin for comparison, demonstrated that the presence of a methoxyl group in C6 of the A ring of flavonol patuletin enhanced its anticancer potential [28]. Yu et al. have purified polysaccharides from the stem extract of the medicinal plant *Dendrobium officinale* grown under different planting conditions (in the greenhouse and in the wild) and compared their structure and antitumor properties on HeLa cells [29]. Polysaccharides showed a significant activity only after oxidative degradation to smaller molecular weight species. The fractions from wild plants showed an evident antiproliferative and pro-apoptotic activities while the effects of the fractions from greenhouse plants were not significant [29]. Nguyen et al. have biotransformed three selected anthraquinones into their O-glucoside by a bacteria glycosyltransferase, and tested these products for their antiproliferative affects against various cancer cells (AGS: gastric; HeLa: cervical; Hep-G2: liver) [30]. They found that the glycosylated derivatives were more effective in inhibiting cell growth than their parental aglycones [30].

Kahnt et al. have synthesized 28 new cytotoxic agents starting from the naturally occurring triterpenoids betulinic and ursolic acid [31]. Different ethylenediamine derived carboxamides were tested for cytotoxicity by the sulforhodamine-B colorimetric assay in several tumor cell lines (518A2: melanoma; A2780: ovarian carcinoma; HT29: colon adenocarcinoma; MCF-7: breast adenocarcinoma; 8505C: thyroid carcinoma) and in nonmalignant mouse fibroblasts (NIH 3T3). Two betulinic acid-derived compounds were identified as the most effective with an EC_50_ lower than 1 µM [31]. Unfortunately, these compounds were not selective for tumor cells since they were toxic also toward nonmalignant fibroblasts. Ling et al. have screened a natural product library containing fractions and pure compounds for proliferation inhibition in different cancer cell models [32]. They identified different alkaloid compounds with a potent cytotoxic effect. In particular, homoharringtonine showed an EC_50_ lower that 0.1 µM and together with cephalotaxine, demonstrated potent inhibition of protein synthesis [32]. Lim et al. have demonstrated an antimelanoma effect of bee venom (BV) and that the major active ingredient is melittin, an amphiphilic peptide containing 26 amino acid residues [33]. These effects were mediated by the downregulation of PI3K/AKT/mTOR and MAPK signaling pathways [33]. 

Three new isochromanes were isolated from *Aspergillus fumigatus* fermentation broth and tested in vitro for their cytotoxic effects by MTT assay of MV4-11 cell line [34]. Only two of them showed a moderate growth inhibition with IC_50_ values of 23.95 and 32.70 µM, respectively [34]. Similarly, four new pentacyclic triterpene were isolated from hexane extract of *Salacia crassifolia* root wood and tested for their cytotoxic activity against human cancer cell lines using the “NCI-60 cell line screen” [35]. Among them, pristimerin showed selective inhibitory activity towards a variety of human tumor cell lines and it was primarily responsible for the cytotoxic activity of the crude extracts [35].

In this Special Issue, six reviews were included aimed to summarize the antitumoral properties of different compounds isolated from several natural sources [36,37,38,39,40,41]. Liu et al. reviewed the anticancer activities of the compounds porphyran and carrageenan, derived from red seaweed [36]. Possible mechanisms in the anticancer activity of these two polysaccharides were considered along with their possible cooperative actions with other anticancer chemotherapeutics [36]. Wang et al. have reported a mini review on the anticancer activity of the naturally occurring indoloquinoline alkaloids cryptolepine, neocryptolepine, and isocryptolepine, isolated from the roots of *Cryptolepis sanguinolenta* and several of their analogues [37]. They presented an overview of the potential of neocryptolepine and isocryptolepine as scaffolds for the design and development of new anticancer drugs [37]. Yang et al. have reviewed diverse in vitro and in vivo pharmacological properties of capsazepine, a synthetic analogue of capsaicin (the common pungent ingredient of hot chili peppers) [38]. In addition to having an anticancer activity, capsazepine has important anti-inflammatory effects reducing the level of some inflammatory mediators [38]. Liskova et al. provided a comprehensive review of studies focusing on the anticancer effectiveness of dietary phytochemicals, either isolated or as mixtures, which act via targeting cancer stem cells (CSCs) [39]. Among dietary compounds able to target CSCs and some of their abnormally activated signaling pathways, epigallocatechin-3-gallat, resveratrol, genistein, curcumin, isothiocyanates, and diallyl trisulfide have been of particular interest [39]. Girisa et al. have considered and reviewed the potential anticancer activity of zerumbone, a sesquiterpene compound isolated from *Zingiber zerumbet* Smith [40], while Choi has reviewed the anti-inflammatory and anticancer activities of phloretin, a chalcone polyphenol present in apple [41].

Natural products are attractive sources for the development of new medicinal and therapeutic agents. Those with antitumoral potential may be more selective and have weaker adverse effects compared to conventional chemotherapy drugs actually used for cancer treatment. Clinical trials are necessary to demonstrated whether the in vitro and in vivo animal data are reproduced in human, and to allow the application of natural products in cancer prevention and treatment.
